# Small Pontine Infarction Secondary to Posterior Inferior Cerebellar Artery (PICA) Atherosclerosis: The Diagnostic Value of 3 Tesla MRI

**DOI:** 10.7759/cureus.103533

**Published:** 2026-02-13

**Authors:** Dang Nhat Vo, Tri Dung Ton That, Vy Duyen Duong

**Affiliations:** 1 Neurology and Stroke Department, Vinmec Da Nang International Hospital, Vinmec Healthcare System, Da Nang, VNM; 2 General Nursing Department, Da Nang University of Medical Technology and Pharmacy, Da Nang, VNM

**Keywords:** 3.0t mri, acute ischemic stroke, atherosclerosis, pica, vertigo

## Abstract

Posterior circulation strokes, particularly those involving the posterior inferior cerebellar artery (PICA), often present with nonspecific symptoms, such as vertigo, frequently leading to misdiagnosis as peripheral vestibular disorders. Identifying small lacunar infarcts in these regions requires high-resolution neuroimaging. A 65-year-old female with a history of hypertension and dyslipidemia presented with sudden-onset rotatory vertigo, headache, and right-sided paresthesia (National Institutes of Health Stroke Scale {NIHSS}=2). The 3 Tesla MRI revealed a 5-mm acute ischemic focus in the left pons on diffusion-weighted imaging (DWI) and apparent diffusion coefficient (ADC) sequences. Three-dimensional (3D) time-of-flight (TOF) magnetic resonance angiography (MRA) demonstrated multifocal atherosclerotic stenosis along the left PICA. The patient was managed with dual antiplatelet therapy (DAPT) (clopidogrel 75 mg and aspirin 100 mg), high-intensity statin (rosuvastatin 20 mg), and stringent blood pressure control. Following seven days of treatment, clinical symptoms improved significantly, and the patient was discharged in stable condition. The 3 Tesla MRI is a pivotal tool for detecting small-core infarcts and evaluating detailed atherosclerotic changes in the posterior circulation. Integrating clinical vigilance with advanced imaging facilitates accurate diagnosis and optimizes medical management to prevent recurrent stroke.

## Introduction

Acute vertigo is a common presentation in emergency departments and poses a diagnostic challenge in distinguishing peripheral causes from central etiologies, such as posterior circulation strokes. The posterior inferior cerebellar artery (PICA), a major branch of the vertebral artery, is susceptible to atherosclerosis [1]. Stenosis of the PICA can result in small, discrete infarctions that may be occult on conventional imaging but can cause significant neurological deficits.

## Case presentation

A 65-year-old female patient presented to the emergency department with a sudden onset of severe rotational vertigo and postural instability. She reported a severe headache accompanied by neck stiffness and nape tenderness, radiating to the right side of the neck and right arm. Additionally, she experienced right-sided paresthesia. Her medical history was significant for hypertension, currently managed with amlodipine 5 mg daily, and dyslipidemia.

Upon admission, the patient was conscious and oriented. Vital signs revealed a blood pressure of 170/100 mmHg, a heart rate of 70 beats per minute, and a respiratory rate of 19 breaths per minute. Clinical examination noted that the vertigo intensified with head movement and was associated with nausea. Although the patient reported a "lightness" and tingling sensation in the right side of her body, objective motor testing showed preserved muscle strength (5/5) in all extremities with no focal motor deficits. The National Institutes of Health Stroke Scale (NIHSS) score was 2.

**Figure 1:**
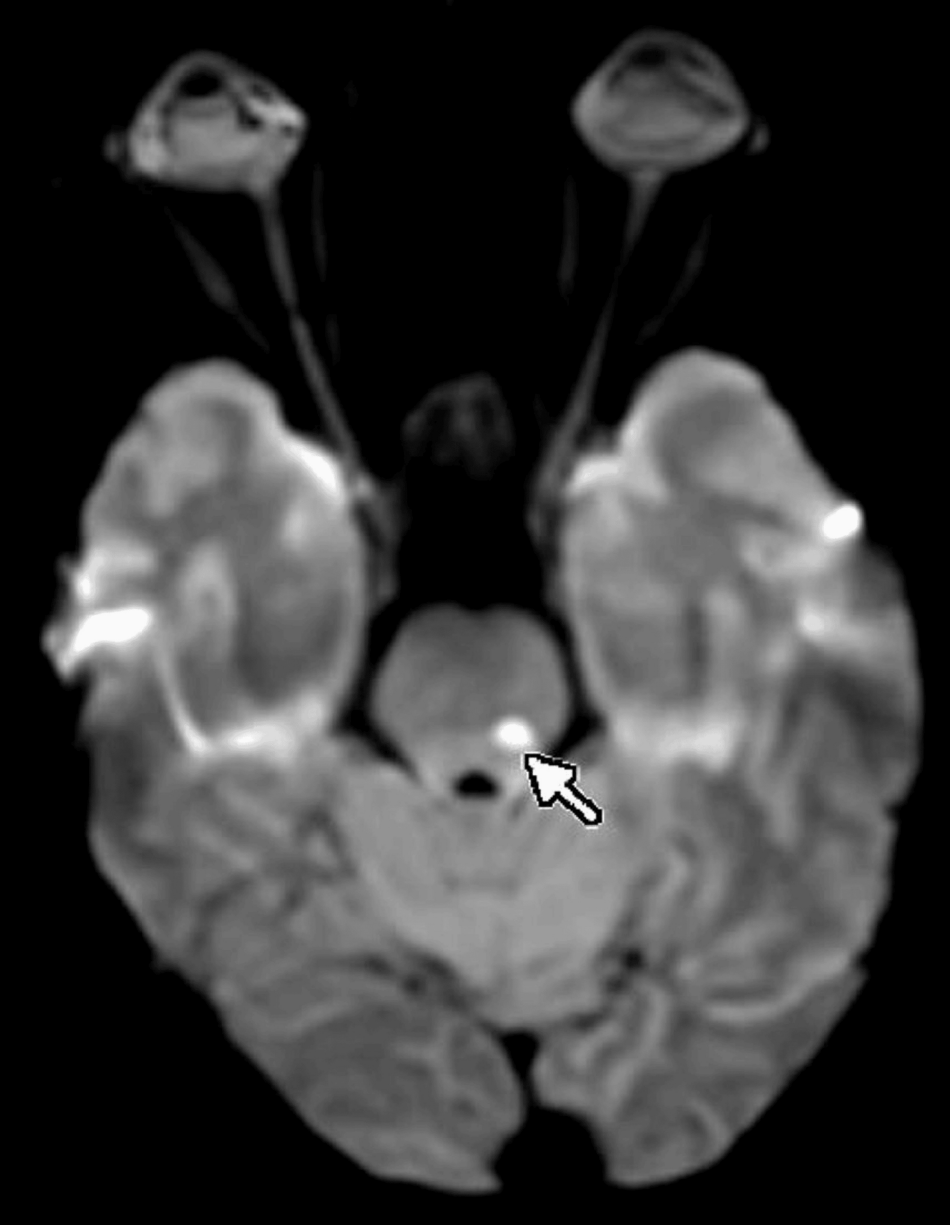
MRI of the brain revealed a high-signal nodule, approximately 5 mm in diameter, located in the left pons on DWI (marked with an arrow). DWI: diffusion-weighted imaging

**Figure 2:**
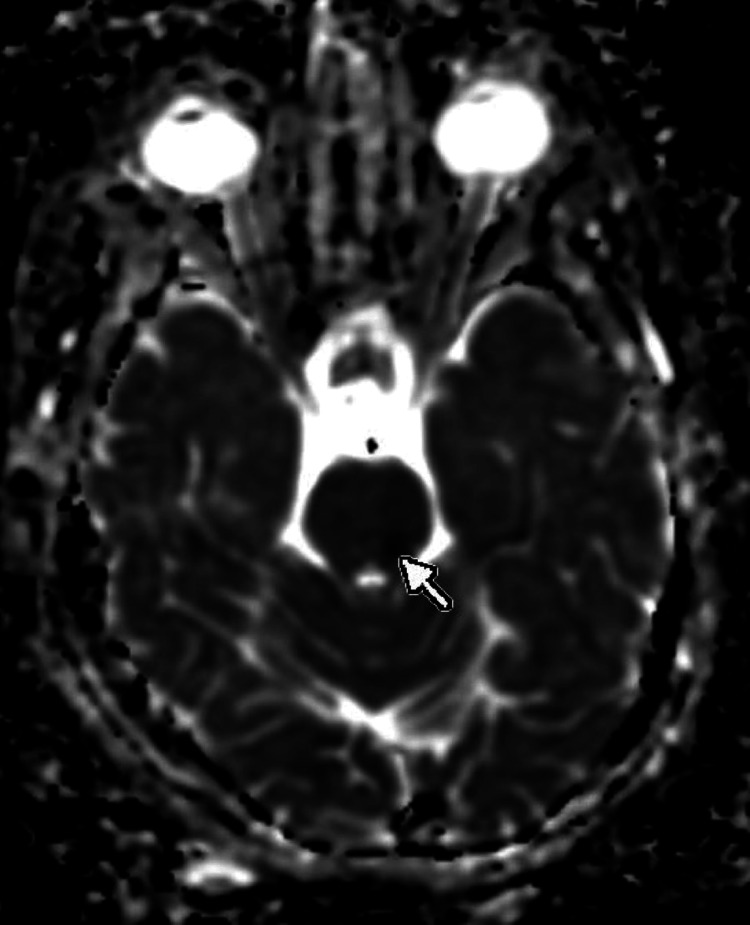
MRI of the brain showed a corresponding hypointense signal, confirming acute diffusion restriction on ADC (marked with an arrow). ADC: apparent diffusion coefficient

**Figure 3:**
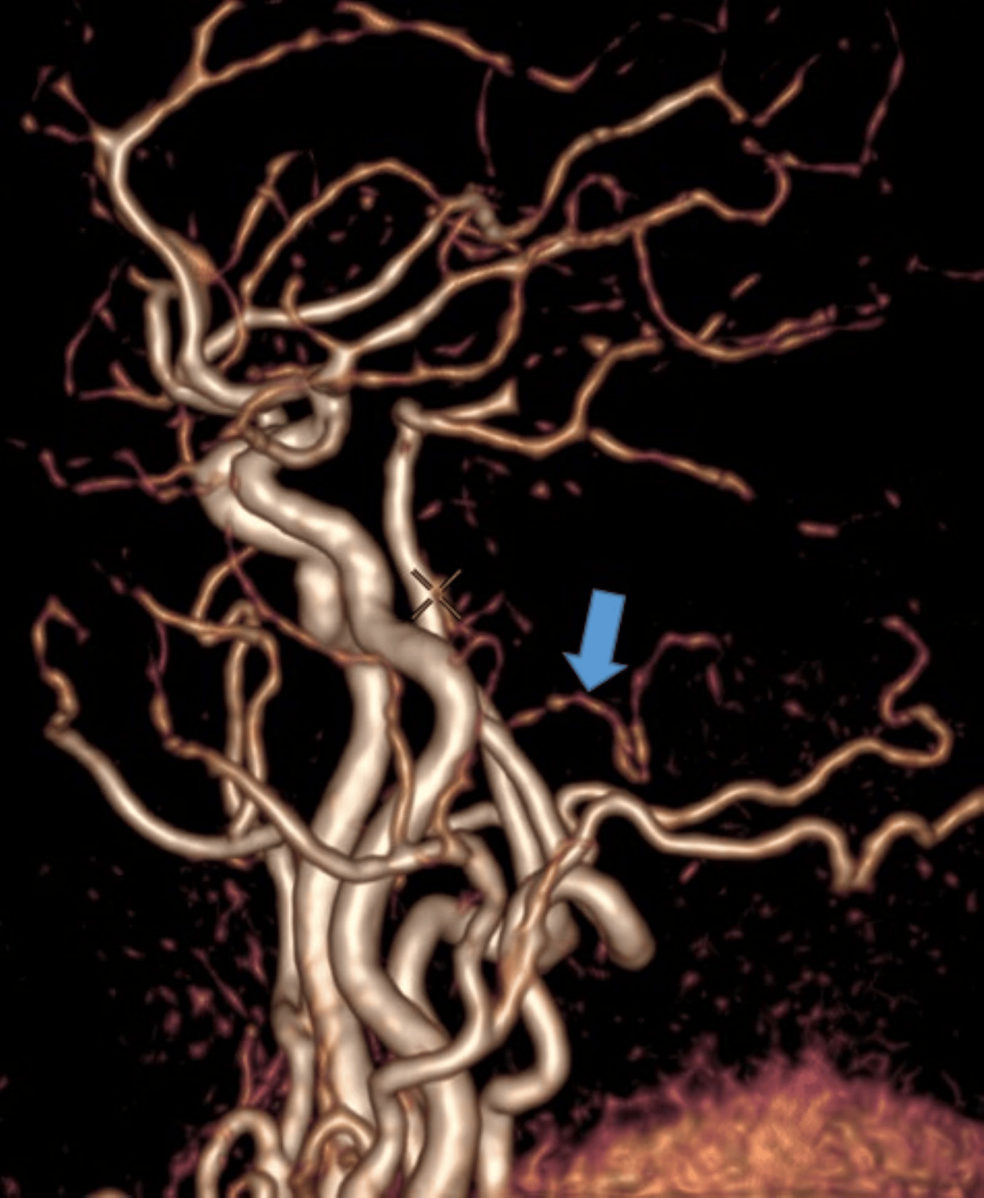
Three-dimensional-TOF MRA visualized multifocal atherosclerotic stenosis throughout the left PICA (marked with an arrow). TOF: time-of-flight; MRA: magnetic resonance angiography; PICA: posterior inferior cerebellar artery

Magnetic resonance imaging (MRI; 3 Tesla {3T}) was performed to investigate the acute neurological symptoms. Diffusion-weighted imaging (DWI) revealed a high-signal intensity nodule, approximately 5 mm in diameter, located in the left pons (Figure 1). The corresponding apparent diffusion coefficient (ADC) map showed signal reduction, confirming restricted diffusion consistent with an acute lacunar infarct (Figure 2). On fluid-attenuated inversion recovery (FLAIR) sequences, the lesion displayed a slight hyperintensity, suggesting an early-stage infarct. Furthermore, 3D time-of-flight (TOF) magnetic resonance angiography (MRA) identified atherosclerotic changes resulting in scattered stenosis at multiple sites along the left posterior inferior cerebellar artery (PICA) (Figure 3). Based on these findings, the patient was diagnosed with an acute ischemic stroke (left pontine infarction), hypertension, and dyslipidemia.

The patient was initiated on an intensive medical management protocol. Dual antiplatelet therapy (DAPT) was prescribed using a fixed-dose combination (DuoPlavin: clopidogrel 75 mg and aspirin 100 mg) once daily. The decision to use DAPT was based on the high recurrence risk associated with large-vessel atherosclerosis and the low NIHSS score of 2. For lipid management, high-intensity statin therapy with rosuvastatin (Crestor) 20 mg daily was administered, targeting a low-density lipoprotein cholesterol (LDL-C) level <1.4 mmol/L (or at least a 50% reduction from baseline) in accordance with guidelines for atherosclerotic stroke. Blood pressure was managed to maintain a target <140/90 mmHg by titrating her amlodipine dosage relative to her acute status.

Following seven days of inpatient treatment in the neurology department, the patient showed significant clinical improvement. The rotational vertigo completely resolved, and she regained the ability to ambulate independently and perform activities of daily living. While some mild right-sided paresthesia remained, the patient was hemodynamically stable upon discharge with a blood pressure of 130/80 mmHg and no new focal neurological deficits.

## Discussion

Clinicopathological correlation and posterior circulation anatomy

The clinical presentation in this case was particularly challenging, as vertigo was the predominant symptom, often mimicking peripheral vestibulopathy. However, the sudden onset of right-sided paresthesia (contralateral to the left pontine lesion) served as the "diagnostic key." In pontine infarctions involving the PICA or perforating branches of the basilar artery, the sensory pathways (lateral lemniscus) and vestibular nuclei are in close anatomical proximity. According to Savitz and Caplan, posterior circulation strokes account for approximately 20% of all ischemic strokes; within this subset, vertigo is the most frequent yet most commonly misdiagnosed symptom [2].

Pathophysiological mechanisms of PICA atherosclerosis

**Figure 4:**
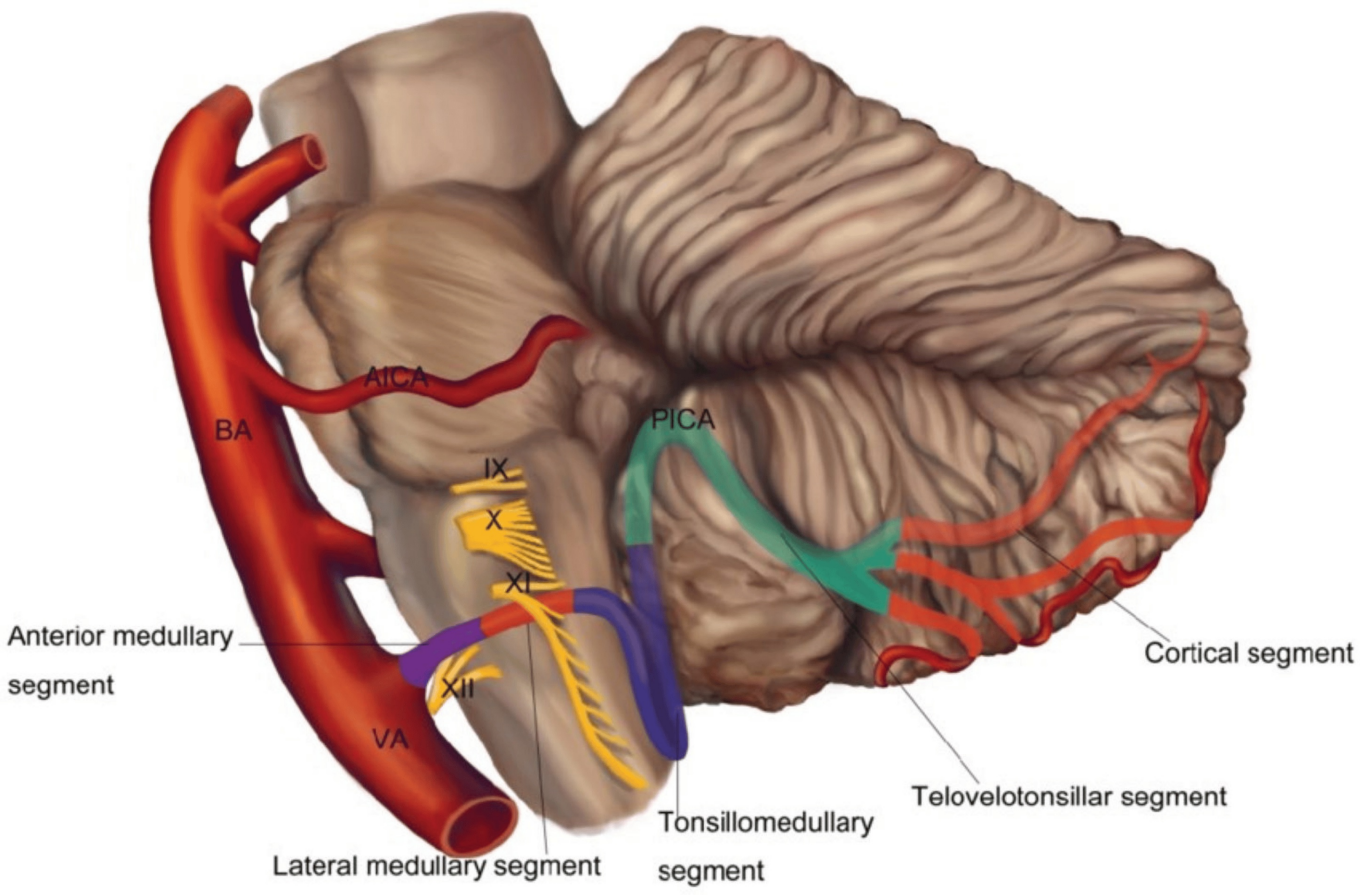
Anatomy and segments of posterior inferior cerebellar artery. The posterior inferior cerebellar artery originates from the vertebral artery, supplying the posterolateral medulla and the inferior part of the cerebellum. Stenosis of the posterior inferior cerebellar artery can lead to localized ischemic events in the brainstem and cerebellar regions. This image has been reproduced from Miao et al. [1], which is an open-access article distributed under the terms of the Creative Commons Attribution License (https://creativecommons.org/licenses/by/4.0/).

The posterior inferior cerebellar artery (PICA) typically originates from the vertebral artery (Figure 4). In this patient, 3D-TOF MRA at 3T MRI identified multifocal atherosclerotic stenosis, consistent with the "artery atherosclerosis" mechanism under the Trial of ORG 10172 in Acute Stroke Treatment (TOAST) classification. Reported intracranial atherosclerotic stenosis (ICAS) prevalence varied widely within the Asian community, from 3% to 89.4% (median=13%), while frequency in ischemic stroke ranged from 7.9% to 82.4% (median=41.65%) [3]. PICA stenosis does not only cause localized ischemia; it can also compromise flow to the perforating branches supplying the posterolateral pons, resulting in the 5 mm lacunar infarct core observed on DWI. The clinical "penumbra" was evidenced by persistent, severe vertigo, suggesting functional impairment of the left cerebellar hemisphere even before a complete necrotic core formed on fluid-attenuated inversion recovery (FLAIR) sequences [4,5].

The diagnostic value of 3 Tesla MRI technology

The sensitivity of MRI in detecting early-stage cerebral infarction significantly surpasses that of CT, particularly in the posterior fossa, where CT is often limited by bone hardening artifacts. The 3T magnetic field provides a superior signal-to-noise ratio (SNR), enabling the visualization of lacunar infarcts only a few millimeters in diameter. Notably, 3T 3D-TOF MRA allows high-resolution reconstruction of small caliber vessels, such as the PICA or anterior inferior cerebellar artery (AICA), with sufficient detail to identify atherosclerotic plaques that previously necessitated invasive procedures like digital subtraction angiography (DSA) [6].

Rationale for dual antiplatelet therapy (DAPT)

The administration of DuoPlavin (fixed-dose clopidogrel+aspirin) in this case aligns with evidence from the Clopidogrel in High-Risk Patients With Acute Non-Disabling Cerebrovascular Events (CHANCE) and Platelet-Oriented Inhibition in New TIA and Minor Ischemic Stroke (POINT) clinical trials [7]. These studies demonstrate that for patients with minor stroke (NIHSS ≤3) or high-risk transient ischemic attack (TIA), DAPT initiated in the acute phase (typically for the first 21-90 days) significantly reduces the risk of recurrent stroke compared to monotherapy, without a substantial increase in major hemorrhage risk. In this patient, the presence of PICA stenosis on 3T MRI further reinforced the indication for DAPT to stabilize the unstable plaque.

Role of high-intensity statin therapy

The use of rosuvastatin 20 mg extended beyond mere lipid-lowering, leveraging the "pleiotropic effects" of statins. Based on the Stroke Prevention by Aggressive Reduction in Cholesterol Levels (SPARCL) trial findings, high-intensity statins contribute to plaque stabilization, exert anti-inflammatory effects, and improve endothelial function. These benefits are critical when 3D-TOF MRA confirms disseminated atherosclerosis within the posterior circulation [8].

Prognosis and long-term management

The short-term prognosis for this patient is highly favorable due to the small infarct volume and timely intervention. However, given the established atherosclerotic disease in the vertebrobasilar system (PICA), the long-term prognosis depends heavily on strict adherence to risk factor modification, specifically blood pressure and lipid management, and the maintenance of a healthy lifestyle to prevent future major vascular events.

## Conclusions

Posterior circulation strokes present with highly diverse clinical manifestations, often leading to potential misdiagnosis. This case underscores the critical importance of maintaining a high index of clinical suspicion when encountering acute vertigo associated with even subtle focal neurological signs, such as isolated paresthesia. The integration of 3T MRI technology represents a significant diagnostic advancement, enabling the precise identification of small-core lacunar infarcts and detailed evaluation of posterior circulation vasculopathy, specifically PICA atherosclerotic stenosis. Such high-resolution imaging is instrumental in guiding timely and aggressive medical interventions, including dual antiplatelet therapy and high-intensity statins, while optimizing risk factor control. Ultimately, this comprehensive approach is essential for preventing major, debilitating stroke events in the future.
